# Prospective cohort study on hospitalised patients with suspected urinary tract infection and risk factors por multidrug resistance

**DOI:** 10.1038/s41598-021-90949-2

**Published:** 2021-06-07

**Authors:** Victor Garcia-Bustos, Ana Isabel Renau Escrig, Cristina Campo López, Rosario Alonso Estellés, Koen Jerusalem, Marta Dafne Cabañero-Navalón, Victoria Morell Massó, Ignacio-Antonio Sigona-Giangreco, José Miguel Sahuquillo-Arce, Iván Castro Hernández, Miguel Salavert Lletí

**Affiliations:** 1 Department of Internal Medicine, University and Polytechnic Hospital La Fe, Avinguda Fernando Abril Martorell, 106, 46026 Valencia, Spain; 2 Department of Internal Medicine, Manises Hospital, Manises, Spain; 3 Department of Internal Medicine, General Hospital La Mancha Centro, Alcázar de San Juan, Ciudad Real Spain; 4 Infectious Disease Unit, University and Polytechnic Hospital La Fe, Valencia, Spain; 5 Department of Microbiology, University and Polytechnic Hospital La Fe, Valencia, Spain

**Keywords:** Microbiology, Risk factors, Urology, Diseases, Infectious diseases, Urogenital diseases

## Abstract

Urinary tract infections (UTIs) are among the most common bacterial infections and a frequent cause for hospitalization in the elderly. The aim of our study was to analyse epidemiological, microbiological, therapeutic, and prognostic of elderly hospitalised patients with and to determine independent risk factors for multidrug resistance and its outcome implications. A single-centre observational prospective cohort analysis of 163 adult patients hospitalized for suspected symptomatic UTI in the Departments of Internal Medicine, Infectious Diseases and Short-Stay Medical Unit of a tertiary hospital was conducted. Most patients currently admitted to hospital for UTI are elderly and usually present high comorbidity and severe dependence. More than 55% met sepsis criteria but presented with atypical symptoms. Usual risk factors for multidrug resistant pathogens were frequent. Almost one out of five patients had been hospitalized in the 90 days prior to the current admission and over 40% of patients had been treated with antibiotic in the previous 90 days. Infection by MDR bacteria was independently associated with the previous stay in nursing homes or long-term care facilities (LTCF) (OR 5.8, 95% CI 1.17–29.00), permanent bladder catheter (OR 3.55, 95% CI 1.00–12.50) and urinary incontinence (OR 2.63, 95% CI 1.04–6.68). The degree of dependence and comorbidity, female sex, obesity, and bacteraemia were independent predictors of longer hospital stay. The epidemiology and presentation of UTIs requiring hospitalisation is changing over time. Attention should be paid to improve management of urinary incontinence, judicious catheterisation, and antibiotic therapy.

## Introduction

Urinary tract infections (UTIs) are among the most common bacterial infections and a frequent cause for hospitalization^1^, especially in the elderly, where they are the most frequent infection requiring hospital admission^2–4^.

Aetiology of UTIs is diverse, but the most frequently isolated organisms worldwide are uropathogenic *Escherichia coli (UPEC)*, *Klebsiella pneumoniae*, *Staphyloccocus saprophyticus*, *Enterococcus faecalis*, Group B *Streptococcus*, *Enterobacter* spp., *Proteus mirabilis*, *Pseudomonas aeruginosa*, *Staphylococcus aureus* and *Candida* spp^5^.

Although bacterial colonization of the lower urinary tract is common, the development of clinically relevant infection results from a combination of both host-specific risk factors (such as diabetes mellitus, obesity, immunodeficiency, previous urinary tract infections, use of indwelling catheters or pre-existing urinary lithiasis) and pathogen-specific risk factors (adhesins, increased survivability against host defences or presence of escape mechanisms, invasiveness, biofilm formation and antibiotic resistance)^6^. Many UTI risk factors and UTI itself are associated with frailty in older adults^7^. Clinical manifestations range widely from asymptomatic bacteriuria to septic shock. Symptomatic UTIs have been either classified as uncomplicated or complicated, depending on whether anatomical or functional abnormalities of the urinary tract are present. Also, patients can be affected by adverse effects of both appropriate and inappropriate antibiotic treatments, including *Clostridioides difficile* colitis and increasing bacterial antibiotic resistance patterns^3^. Additional complications of UTIs have been described, such as an increased risk for stone formation in the urine tract, persisting renal insufficiency or, in pregnant women, preterm delivery^3,4,6,8^.

Diagnosis of UTIs is based on the history and clinical features combined with results of urine biochemical analysis and urine culture. Results of bacterial cultures are not usually available within 48 h, which means that for symptomatic UTIs, antibiotic treatment is usually prescribed empirically^8,9^.

Recently, increasing antibiotic resistance patterns are being reported, highlighting the need for health care providers to analyse local antibiotic resistance patterns in order to adequately guide these empirical treatments^10^. Ideally, physicians should adapt empirical antibiotic therapy to known local bacterial flora and resistance patterns. Different bacterial flora can be found among hospitals within the same region, and even among different floors within the same building. Thus, epidemiological surveillance and reporting is necessary in order to guide antibiotic stewardship, which has shown to significantly reduce duration of antibiotic therapy and related costs without worsening treatment efficacy or prognosis^11,12^. Furthermore, UTI epidemiology is changing over time, with increased comorbidity, age, polypharmacy and instrumentation of the urinary tract, and up-to-date data are scarce^13–15^.

In elderly patients, UTI is a major cause of functional deterioration. It leads to increased vulnerability, and frailty, and decreases the chances of recovery of basic independent activities.

The aim of our study was to analyse epidemiological, microbiological, therapeutic and prognostic data in a prospective cohort of elderly hospitalised patients with UTI in a tertiary care centre in Valencia, Spain, and to determine independent risk factors for multidrug resistance and its outcome implications.

## Materials and methods

### Study design and setting

Our study is a single-centre observational cohort analysis of adult patients hospitalized for suspected symptomatic UTI at the time of admission in the Departments of Internal Medicine, Infectious Diseases and Short-Stay Medical Unit of the University and Polytechnic Hospital La Fe (UPHLF) of Valencia (Spain), in the 2-year period between January 2014 and January 2016. The study protocol was approved by the Ethics Committee of the Health Research Institute La Fe, being assessed under the internal registration code 2014/0668, and was compliant with the Declaration of Helsinki. The study is in accordance the STROBE guidelines, and the checklist is provided in Supplementary file 1.

UPHLF is a university hospital of 996 beds providing tertiary care in Valencia, Spain, and has an assigned population of 300,000. It is the hospital of reference of the Valencian Community (approximate population, 5,000,000).

### Definitions and variables

Patients who presented to the emergency department (ED) or other hospital ward prior to admission in our hospital with a history, examination, and urine sediment compatible with cystitis, prostatitis, pyelonephritis, or urinary sepsis were selected. All patients admitted with suspected or microbiologically confirmed UTI were included. The diagnostic criteria in our institution followed the Guidelines for the diagnosis and treatment of urinary tract infection of the Spanish Society of Clinical Microbiology and Infectious Diseases (SEIMC). Patients with suspected ITU were defined as those with history, clinical symptoms, physical examination, and urine sediment compatible (namely, significant pyuria and/or positive nitrites) compatible with cystitis, prostatitis, pyelonephritis, or urinary sepsis, in absence of a positive urine culture in properly obtained samples and concentration. Confirmed UTI was defined with previous criteria and significant isolation of microorganisms in urine, as follows:

- Growth of ≥ 10^5^ colony forming units (CFU)/mL of a single isolate.
- Growth of ≥ 10^5^ colony forming units (CFU)/mL with mixed growth but one predominant microorganism.
- In pyelonephritis, growth of ≥ 10^4^ CFU/mL of a single isolate.
- Growth of ≥ 10^3^ CFU/mL of a single isolate in men.
- Growth of ≥ 10^2^ CFU/mL of a single isolate in strongly symptomatic women.
- Growth ≥ 10^2^ CFU/mL of a single isolate in urine obtained from urinary catheter.
- Any growth in urine obtained from suprapubic aspiration.

Significant asymptomatic bacteriuria was defined as follows:

- Growth of ≥ 10^5^ CFU/mL of the same isolate in two samples or in one sample + positive nitrites in urinary sediment of other sample in women.
- Growth of ≥ 10^5^ CFU/mL of a single isolate in one sample in men or in urine obtained from urinary catheter.

Retrospectively correct empirical antibiotic choice was defined as follows:

- Clinical and analytical resolution of the episode after first-choice empirical antibiotic therapy.
- Adequate antibiotic coverage with the elected empirical antibiotic treatment according to the sensitivity profile of the causative microorganism shown by the antibiogram of the isolate in the urine culture.

Signed informed consent to access patient data was obtained at the time of enrolment and they were followed prospectively. A 6-month follow-up was made.

Included variables were virtually divided into different sections: demographics, comorbidities, risk factors for UTI and multidrug resistant (MDR) organisms, clinical, analytical, and microbiological parameters, and treatment and outcomes.

Basic demographic data, residency before hospitalisation and dates of admission and discharge (or death) were recorded.

Individual comorbidities (previous urine tract abnormalities, diabetes mellitus (DM), obesity, cardiovascular disease (CVD), chronic obstructive pulmonary disease (COPD), chronic liver disease (CLD), active oncological illness, immunosuppressive states such as transplant carriers, HIV + status and malnutrition), as well as Charlson Comorbidity Index (as a predictor of 10-year survival depending on comorbidities) and Barthel Index (for assessing functional independence) were also analysed. In the Barthel Index, 0–19 points were interpreted as complete dependence and 80–100 points as independence. Both questionnaires and their interpretation are included in Supplementary file 2.

Pre-existing urinary tract obstruction, vesicoureteral reflux, urine tract lithiasis, benign prostate hyperplasia (BPH), bladder cancer, catheters or foreign material in the urine tract, paraplegia, tetraplegia or a prolonged bedridden state, previous UTIs and sexually transmitted diseases (STDs), neutropenia and urinary or faecal incontinence, were considered to be predisposing risk factors for UTIs.

Previous suspected or confirmed UTIs and STDs, hospitalization or antibiotic treatments 90 days prior to admission, antibiotic treatment in the last 90 days before admission or healthcare associated origin of UTI were recorded as risk factors for MDR organisms. MDR was defined as acquired non-susceptibility to at least one agent in three or more antimicrobial categories^16^.

Clinical, analytical and microbiological data at the time of admission included: sepsis criteria, fever or hypothermia, hypotension and need for vasopressor treatment, tachypnoea, tachycardia, altered mental status, leukocytosis, decreased renal function, C-reactive protein (CRP), procalcitonin (PCT), lactate, urinary sediment analysis, dates and results of blood and urine cultures, bacterial count and antimicrobial resistance patterns. Records of previous positive culture from the same patients were also assessed.

Initial empirical antibiotic treatment and further modifications and dates were recorded. Outcomes, including curation, recurrence, need for rehospitalization and 6-months survival were assessed, as frailty indicators after a UTI.

### Statistical analysis

The statistical analyses were conducted with R statistical software version 4.0.1 (R Development Core Team, 2017, Vienna, Austria). Quantitative data were expressed as mean and standard deviation (SD), whilst qualitative data were expressed as absolute count and percentage of cases. Normality and homogeneity of variance were assessed with quantile–quantile plots and Levene test, respectively. Two-tailed p-value below 0.05 was considered statistically significant. T-test and ANOVA test were used for mean comparison in two or more groups, respectively. In the comparison of qualitative variables, chi-square test was used. On the one hand, a multivariable logistic regression model was performed to determine risk factors for isolation of multidrug resistant organisms. On the other hand, a multivariable linear regression model was built to identify independent predictors for long hospital stay, both in the complete cohort and in those patients with positive urine culture.

Variable selection was carried out firstly by including variables according to known evidence and biological plausibility and, lately, by means of a stepwise Akaike information criterion (AIC) method in order to select those variables to include in the final model. The discrimination of the final simplified logistic regression model was assessed by means of the receiver operating curve (ROC) and the area under the curve (AUC). Odds ratios (OR) and 95% confidence intervals (95% CI) were calculated.

## Results

### Patients’ characteristics and comorbidities

A total of 163 patients were included in the study, 79 (48.5%) of them were male and 84 (51.5%) were female. Their mean age was 78.93 (14.8) years and significantly differed among departments (p < 0.0001). The mean age was 77.15 (14.87), 83.02 (10.75) and 57.10 (16.19) in the Departments of Internal Medicine, Infectious Diseases and Short-Stay Medical Unit, respectively. Approximately 82% (135) of UTIs were community-acquired, while 17.79% (28) were considered healthcare-associated infections.

The mean Charlson Comorbidity Index in our population was 4.9 (± 1.99), which estimates a 10-year predicted mortality of almost 20%. The mean Barthel index in our cohort was 48.1 (± 36.0), indicating partial dependence. Both indexes were significantly different between departments (p < 0.0001). Patients were more comorbid and more dependent in the Short-Stay Medical Unit, followed by Internal Medicine Department and Infectious Diseases Unit (Charlson index mean of 5.27 [± 1.76], 4.81 [± 2.04], 2.8 [± 1.99], respectively; Barthel index mean of 40.09 [± 33.06], 70.46 [± 34.4], 65.95 [± 37.59], respectively). The most frequent comorbidities in our study population were urinary incontinence (49.69%), followed by cardiovascular disease (45.4%), diabetes mellitus (40.49%), prolonged bedridden state (31.29%), BPH (25.15%), faecal incontinence (25.15%) and chronic kidney disease (21.47%). Thirty-one patients had an active neoplasia. Of these, 20% were prostate tumours, followed by uterus neoplasia (16.6%), haematological malignancies (13.3%) and colon cancer (10%). Additionally, over 22% of UTIs were associated with intermittent or permanent urinary catheterization. Of our 163 patients, 81 had a history of previous UTIs. Nearly 24% had previously been hospitalized and more than 40% had received antibiotic therapy 90 days prior to hospital admission. Further details are represented in Table 1.

**Table 1:** Population comorbidities.

Variable	Mean (SD)/n (% of total)	N included in the analysis
Mean Charlson index	4.9 (1.99)	163
Mean Barthel Index	48.1 (36.02)	163
Chronic kidney disease	35 (21.47)	163
Urinary tract obstruction	10 (6.13)	163
Hypertrophic prostate disease	41 (25.15)	79
Nephrolithiasis	9 (5.52)	163
Vesicoureteral reflux	5 (3.07)	163
Permanent bladder catheter	26 (15.95)	163
Intermittent bladder catheter	10 (6.13)	163
Ureteric (double J) stent	3 (1.84)	163
Nephrostomy	0 (0)	163
Other urine tract abnormalities	13 (7.98)	163
Diabetes mellitus	66 (40.49)	163
COPD	16 (9.82)	163
Cardiovascular disease	74 (45.4)	163
Chronic liver disease	10 (6.13)	163
Neoplasm	31 (19.02)	163
Transplant recipient	1 (0.61)	163
HIV +	8 (4.91)	163
Obesity	20 (12.27)	163
Malnutrition	15 (9.2)	163
Prolonged bedridden state	51 (31.29)	163
Paraplegia or tetraplegia	10 (6.13)	163
Urinary incontinence	81 (49.69)	163
Faecal incontinence	41 (25.15)	163
Previous UTI	81 (49.69)	163
Previous recorded STD, non-HIV	1 (0.61)	163
Hospitalization 90 days prior	39 (23.93)	163
Antibiotic use 90 days prior	66 (40.49)	163

### Clinical and laboratory parameters

At the time of admission, more than half of the patients met sepsis criteria, according to the Third International Consensus for Sepsis and Septic Shock 2016, and 8 patients (4.9%) presented with septic shock. Six patients received vasopressor treatment in the hospital ward. Elevated heart rate was the most frequently altered vital sign (50%). Whilst mean body temperature was somewhat elevated (37.27 °C), fever over 38.3 °C was seen only in 28.4% of patients. Acute kidney injury (45.4%) and altered mental status (39.26%) dxwere frequently attributed to the UTI.

All patients presented with an abnormal urine sediment, with significant leukocyturia (97.53%) being the most frequently abnormal marker, followed by haematuria (72.22%), bacteriuria (67.9%) and positive nitrites (48.77%). Blood analysis revealed elevated C-reactive protein levels in 84.81% of patients and leukocytosis in 54.6%. When performed, elevated lactate serum concentrations were observed in 51.16%, with a mean of 2.51 (± 1.33) mmol/L, and elevated procalcitonin levels were seen in 41.03% with a mean level of 7.31 ng/mL. More details on clinical and analytical parameters can be seen in Table 2.

**Table 2:** Clinical and analytical characteristics at the time of diagnosis.

Variable	Mean (SD)/n (% of total)	N included in the analysis
Mean systolic blood pressure	127.37 (29.06)	163
Mean diastolic blood pressure	68.07 (14.61)	163
Hypotension	23 (14.11)	163
Mean leukocyte count in nº/mm^3^	14,636.3 (12,730.88)	163
Leukocytosis	89 (54.6)	163
Leukopenia	2 (1.23)	163
Neutropenia	0 (0)	163
Mean body temperature in ºC	37.27 (1.1)	162
Fever	46 (28.4)	162
Hypothermia	7 (4.32)	162
Mean heart rate in beats/minute	91.16 (18.19)	162
Tachycardia	81 (50)	162
Tachypnoea	12 (9.38)	35
Presence of pressure ulcer	22 (13.5)	163
Acute kidney injury	74 (45.4)	163
Altered mental state	64 (39.26)	163
Mean C-reactive protein in mg/dL	118.52 (105.33)	158
Elevated CRP	134 (84.81)	158
Median procalcitonin in ng/mL (1st–3rd quartile)	0.58 (0.008–2.25)	39
Elevated procalcitonin	16 (4.03)	39
Mean lactate levels in mmol/L	2.51 (1.33)	86
Elevated blood lactate	44 (51.16)	86
Sepsis criteria	91 (55.83)	163
Need for vasopressors	6 (3.68)	163
Leukocyturia	158 (97.53)	162
Positive urine nitrites	79 (48.77)	162
Bacteriuria	110 (67.9)	162
Haematuria	117 (72.22)	162
Concurring non-urinary bacterial infection	28 (17.18)	163

### Therapeutic and microbiological parameters

All patients received empiric antibiotic treatment, with mean time between presentation and antibiotic initiation being 6.72 (± 4.68) hours. Retrospectively, about 80% of patients received appropriate empiric antibiotic therapy, both initiated in the ED or the day after admission. Mean time between presentation and initiation of appropriate antibiotic was 12.84 (26.38) hours. Fifty-four (33%) patients were treated with combination antibiotic therapy. Antibiotic therapy was changed in 36 (22.1%) patients, and 44 patients further received oral treatment.

Nearly 71% of patients had a positive urine culture result. Of the 46 patients without a positive urine culture result, 18 (11.04%) had no culture collected, in 12 (7.36%) the culture was contaminated, and it was negative in 16 patients (9.81%). In the latter group, 11 patients had previously received oral out-patient antibiotic treatment and in 5 patients the urine culture was taken after several doses of intravenous antibiotherapy during hospitalization. The most frequently found microorganism in our study was *E. coli*, being present in 53 patients, followed by *K. pneumoniae and P. aeruginosa*, which were both present in 11 patients. *E. faecalis* was isolated in 6 patients and there were no *Enterococcus faecium* isolations in our cohort. *Corynebacterium urealyticum* and *Enterobacter cloacae* were present in 3 patients, and *P. mirabilis* was seen in 2 patients. There was only one case of ITU caused by each of the following microorganisms: *S. aureus,* coagulase-negative *Staphylococcus spp.*, *Morganella morgagnii, Klebsiella oxytoca, Corynebacterium jeikeium, Citrobacter freundii.* One case of invasive ITU due to *Candida albicans* also occurred. Nine infections were polymicrobial. Of these bacteria, 57.29% showed some form of acquired antimicrobial resistance. Twenty-one per cent showed extended spectrum beta-lactamase activity (ESBL), of which 4 cases had inhibitor-resistant TEM (IRT) or OXA beta-lactamases, 16.7% showed quinolone resistance, 5 isolated Gram-positive microorganisms were methicillin-resistant -including *Corynebacteriae-*, and 4.2% of cases were resistant to 2 or more classes of antibiotics. Only one patient had previous isolation of carbapenemase-producing microorganisms.

In 93 patients, blood cultures were extracted, 24 of them were positive, 8 were contaminated and 69 were negative. In 78.95% there was concordance between urinary and blood isolates. *E. coli* was also the most frequently isolated organism and was present in 12 blood cultures. Four showed more than one microorganism, 3 were positive for *K. pneumoniae*, 2 were positive por *P. mirabilis* and there was one isolation of each *S. aureus, E. cloacae and E. faecalis.*

MDR was significantly associated with lower cure rates (p = 0.036), with complete resolution of the ITU in 100% of patients with non-resistant organism versus 89.1% in those with MDR organisms. Regarding pathogens, *Corynebacterium spp.* and KES (*Klebsiella, Enterobacter and Serratia)* group *Enterobacteriaceae* were significantly associated with higher failure rates, with 50% and 93% response rates, respectively (p < 0.001). No significant differences were seen in patients treated with monotherapy versus combined treatment. Further information is represented in Table 3.

**Table 3:** Detail on microbiological test and therapeutic parameters.

Variable	Mean (SD) / n (% of total)	N included in the analysis
Previously known asymptomatic bacteriuria	8 (4.91)	163
Positive urine culture	117 (71.77)	145
Polymicrobial infection	9 (6.38)	117
Antimicrobial resistance	55 (33.74)	117
ESBL resistance pattern	28 (17.18)	117
Positive blood cultures	24 (25.81)	93
Same microorganism in urine and blood	15 (78.95)	93
Mean time from presentation to ED until antibiotic treatment initiation, in hours	6.72 (4.68)	157
Empirical antibiotic treatment	163 (100)	163
Mean time from presentation until effective antibiotic, in hours	12.84 (26.38)	157
Mean treatment duration, in days	10.69 (3.84)	162
Retrospectively correct empirical antibiotic choice	129 (80.62)	160

### Outcomes

The mean total hospital stay was 9.39 days (10.92). A multivariable linear regression model was used to identify independent predictors for hospital stay. In the first-step variable selection process, the following variables were included: Barthel index, Charlson Comorbidity Index, presence of sepsis criteria, diabetes mellitus, cardiovascular disease, age, prolonged bedridden state, kidney failure, hours until the initiation of adequate antibiotic therapy, isolation of microorganisms in blood cultures, malnutrition, neoplasm, obesity, prolonged fever, MDR organisms, sex, and permanent urinary catheter. The Charlson Comorbidity Index, presence of positive blood cultures, obesity, and female sex were proven to be independent predictors for longer hospital stay. Further data on the linear regression analysis with the selected variables are detailed in Table 4.

**Table 4:** Linear regression model of risk factors for long hospital stay.

Variable	Estimate (95% CI)	Standard error	t-value	p-value
(Intercept)	0.050 (− 5.59–5.69)	2.840	0.017	0.986
Charlson Comorbidity Index	1.109 (0.15–2.062)	0.480	2.315	0.023
Prolonged bedridden state	− 0.043 (− 3.525–3.43)	1.751	− 0.024	0.980
Positive blood cultures	4.366 (0.60–8.13)	1.894	2.306	0.024
Neoplasm	− 3.839 (− 8.50–0.82)	2.350	− 1.636	0.105
Obesity	7.794 (3.04–12.54)	2.391	3.260	0.001
Female sex	4.374 (0.94–7.80)	1.723	2.539	0.013

The same analysis was performed in the subgroup of patients with confirmed positive urine culture. The variables selected by the model are detailed in Table 5. Barthel index, prolonged bedridden state and female sex were independent predictors of longer hospital stay in this group of patients (Table 5). Most patients (92%) were considered cured of their UTI during the time of admission. Four patients required intensive care unit (ICU) admission. Nineteen patients died during hospitalization (11.6%). Of these deaths, 10 were directly attributable to the UTI. Despite the fact that absolute mortality rate was directly related to the frequency of septic patients, isolation of MDR organisms and the mean time until appropriate antibiotic therapy, these differences were not statistically significant. Over one quarter (25.42%) of patients presented a new UTI in the 6-months period following admission, however, there was only 1 patient discharged with prophylactic antibiotic therapy and merely 8 patients had identified asymptomatic bacteriuria prior to admission. Furthermore, 34 (23.61%) of the patients who survived the ITU episode died in the following 6 months.

**Table 5:** Linear regression model of risk factors for long hospital stay in the subgroup of patients with positive urine tract infection.

Variable	Estimate (95% CI)	Standard error	t-value	p-value
(Intercept)	16.202 (9.69–22.71)	3.258	4.971	0.000
Barthel index	− 0.101 (− 0.16 to − 0.04)	0.030	− 3.276	0.001
Charlson Comorbidity Index	− 1.350 (− 2.797–0.10)	0.724	− 1.865	0.066
Prolonged bedridden state	− 6.849 (− 11.50 to − 2.12)	2.327	− 2.942	0.004
Positive blood cultures	2.024 (− 1.72–5.77)	1.875	1.079	0.284
Obesity	4.047 (− 0.69–8.79)	2.372	1.706	0.092
Female sex	4.453 (0.96–7.95)	1.747	2.5486	0.013

### Risk factors for MDR organisms

**Figure 1:**
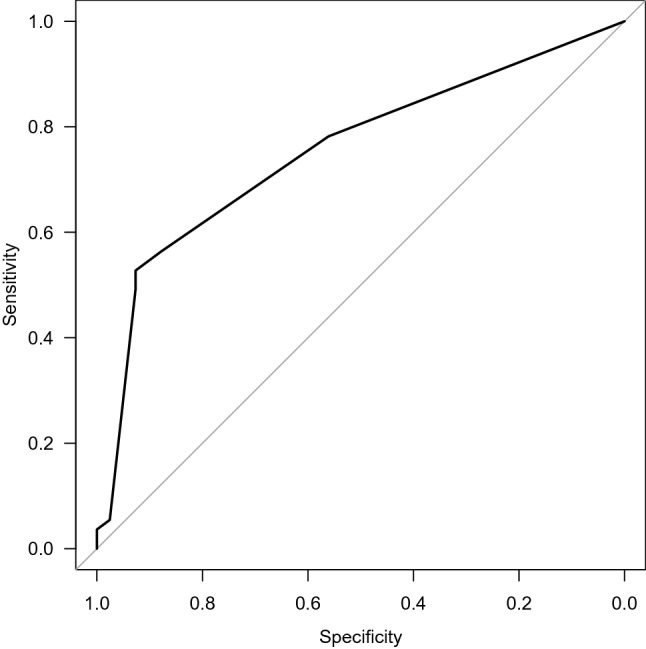
Area under the receiver operator curve of the regression model determining the risk factors for MDR organisms in UTI hospitalised patients.

A multivariable logistic regression analysis was performed to determine risk factors for MDR organisms in our cohort. In a first-step variable selection process, the following parameters were included (age, prolonged bedridden state, diabetes mellitus, urinary tract anomalies, chronic kidney disease, cardiovascular disease, malnutrition, neoplasm, urinary incontinence, faecal incontinence, previous stay in long-term care facilities (LTCF) or nursing homes, hospital admission in the previous 90 days, antibiotic therapy in the previous 90 day and previous UTI). The final model was constructed with the parameters selected by the stepwise AIC method of the previously included variables. Details of the final model are represented in Table 6. Infection by MDR bacteria was independently associated with the previous stay in nursing homes or long-term care facilities (OR 5.8, 95% CI 1.17–29.00), permanent bladder catheter (OR 3.55, 95% CI 1.00–12.50) and urinary incontinence (OR 2.63, 95% CI 1.04–6.68). However, recent antibiotic therapy or hospital admission, and other comorbidities were not independent risk factors in our cohort. The model showed acceptable discrimination, with an area under the curve (AUC) of 0.754 (95% CI 0.66–0.848). The ROC curve is represented in Fig. 1.

**Table 6:** Logistic regression model of risk factors for UTI caused by MDR organisms.

Variable	Estimate	Standard error	OR (95% CI)	Z-value	p-value
(Intercept)	− 0.7668	0.345	0.465 (0.24–0.91)	− 2.222	0.0263
Urinary Incontinence	0.9526	0.476	2.63 (1.04–6.68)	2.002	0.0453
LTCF or nursing homes	2.0036	0.803	7.42 (1.17–29.00)	2.496	0.0126
Permanent urinary catheterisation	1.2762	0.637	5.8 (1.00–12.50 =	2.004	0.045

## Discussion

This study has shown that most patients currently admitted to hospital for UTI are elderly and usually present high comorbidity and severe dependence. In our population, usual risk factors for multidrug resistant pathogens were frequent^17^. Almost one out of five patients had been hospitalized in the 90 days prior to the current admission and over 40% of patients had been treated with antibiotic in the previous 90 days. Remarkably, urinary incontinence and permanent urinary catheter were seen in 50% and 16% of the patients, respectively. These two factors and previous stay in nursing homes or LTCF were independent risk factors for drug resistant organisms-associated infections. Hence, unadjusted empirical antibiotic therapy could fail to adequately cover UTIs in these patient groups or delay the establishment of an appropriate antibiotic therapy, resulting in increased morbidity and mortality. However, in contrast to evidence provided by other works and systematic reviews, recent antibiotic therapy or hospital admission were not associated with a significant increase in antibiotic resistance in the regression model^17,18^. Dependence, comorbidity, obesity, female sex and bacteraemia, on the contrary, were proven to be related to the length of the hospital stay.

As it is our case, previous reports have estimated that up to 80% of complicated UTIs in the United States are attributable to indwelling catheters^19^. Furthermore, they have been identified as independent risk factors and have been associated with increased morbimortality^20,21^. This gains importance if we consider that in many cases they are unnecessary and almost 50% of associated UTIs could be prevented^22^.

Most infections were severe or complicated and more than 55% met sepsis criteria. However, interestingly less than 30% of the patients presented with fever at the time of admission, what might delay the diagnosis and initiation of antibiotic therapy. Furthermore, UTI diagnosis based on clinical data alone has a high error rate. This study also evidences that frail elderly usually present with atypical clinical signs, such as isolated altered mental status and no fever, what may delay both diagnosis and prompt antibiotic therapy.

In our study, patients were selected by clinical and analytical criteria at the time of hospitalization. Even in this scenario, only 72% of our urine cultures resulted positive. These cultures are essential in order to guide the treating physician towards the most appropriate antibiotic therapy. We identified 18 patients in whom no urine culture samples were drawn, neither in the emergency department nor during admission, and the urine culture was contaminated in 12 patients. Despite all patients were admitted with clinical and analytical signs of infection with significant pyuria and/or presence of nitrites and in absence of other infectious causes, possibly no further cultures were taken by their responsible physicians due to good clinical evolution. In 16 patients, the urine culture was negative, what prompts to clarify whether a misdiagnosis was made, especially as some diseases such as interstitial cystitis or painful bladder syndrome may mimic UTI. However, these patients presented with analytical findings of infection (e.g., elevated C-reactive protein or procalcitonin, significant leucocytosis) in absence of other infectious origin, which would be absent in the latter. When reviewing the data, this group of patients had previously received empirical antibiotic therapy, what might have cleared the bacteriuria. These findings emphasize the need for urine cultures before initiating antibiotic therapy especially in susceptible patients with risk factors for adverse outcome or hospital admission in order to make a correct diagnosis and ensure adequate antibiotic treatment in the era of the resistance.

Risk factors for longer hospital stay, both in the whole cohort and in those patients with confirmed urinary culture, were similar. Important comorbidity and dependence and, interestingly, female sex and obesity, were predictors of longer hospitalisation. Besides, in the whole cohort, bacteraemia was also associated to longer stays. However, data in the subgroup with positive cultures are limited due to lower sample size and an increased probability of type 2 error must be acknowledge, as it could happen with the variable obesity.

Despite the fact that more than half of the patients had suffered a previous episode of both confirmed or suspected UTI 90 days prior to admission, only 8% had previous documented significant asymptomatic bacteriuria. In concordance with previous reports, *E. coli* was the most frequent isolated organism at the time of diagnosis, followed by *K. pneumoniae* and *P. aeruginosa. Enterococcus spp.* were less frequent in our series, and no resistant enterococci were observed. This contrasts with previous literature evidencing that enterococci are approximately the second most frequently isolated pathogens in complicated UTIs and present a rising pattern of resistance^3,23^. Drug resistance was observed in 33.7% of the patients. Nevertheless, in almost 80% of the patients the initial empirical antibiotic therapy was optimal, and it was further changed or de-escalated in 22.1% of the patients. In this series, antibiotic therapy was initiated remarkably late, possibly due to the absence of fever and vague clinical symptoms and signs in elderly patients with significant comorbidity.

Summarizing, most patients suffering UTI tend to be highly comorbid, frail, and dependent, and the frequency of MDR organisms is also remarkable, especially in those in nursing homes or LTCF. Acute events such as UTI lead to a vicious circle of increased frailty, which in turn increases further risks of morbidity and mortality.

Interestingly and as it has been shown in other works, no antibiotics and deferred antibiotics were associated with a significant increase in bloodstream infection and all-cause mortality compared with immediate or early initiated antibiotics^4,13^. Similar to other Spanish cohorts, the all-cause mortality rate in our cohort was 11%^24^, whilst Eliakim et al. reported a mortality rate of almost 9% in their multicentre retrospective cohort study^13^. However, the mortality rate widely differs among series, ranges between 3 and up to 33% in some works and may depend on the characteristics of the included patients^13–18,25–27^. In our cohort, the fragile state of the older population manifested as severe dependence was significantly associated to longer hospital stay as previously reported^28^. Nevertheless, Barthel index was not clearly associated to higher mortality rates and no independent predictors of mortality were identified in the regression analysis, probably due to low sample size and a small number of acute events. Despite this, mortality rates seemed to be higher in patients with sepsis, isolation of MDR organisms and in those with higher mean time until appropriate antibiotic therapy. Further cohorts with higher sample size should be performed to confirm these relations.

The presentation of patients with UTI who need hospitalization is changing, and current data are scarce^13^. Most evidence comes from case–control studies or retrospective cohort analyses, but there are very few recent prospective-cohort-studies and, those being, present a rather small sample size^29,30^. The strengths of our study are the prospective inclusion, follow-up and analysis of the cases, the multivariable regression with variable selection based on biological plausibility and stepwise AIC method which avoids confusion bias and high collinearity, the use of management variables not usually emphasized in previous literature as well as the representativeness and generalizability to the Spanish elderly population. However, several limitations are worth mentioning. The relatively small sample size increases the possibility of type II error in the mortality analysis or incidence of less frequent adverse events or outcomes. Besides, assessing the real-life care management includes those limitations of observational studies using routine clinical practice data, such us potential biases of independent physician decisions, missing data, or unmeasured and residual confounders. These problems were addressed by creating a detailed and strict protocol for data collection and measurement variables, which was also shared with the attending physicians of the different departments. Moreover, during the development of this study, an antibiotic use optimization program was running in the three departments.

In conclusion, we found that the epidemiology and presentation of UTIs requiring hospitalisation is changing over time, with patients being older, frail, with severe comorbidity, and suffering severe or complicated infections with vague symptoms that delay the initiation of optimal treatment. Furthermore, we found that urinary incontinence, permanent urinary catheter, and previous stay in nursing homes or LTCF are risk factors for MDR organisms. Our study suggests that the need of indwelling catheters should be reassessed, as many of them might be unnecessary, and prophylactic therapy could be evaluated in those patients with higher risk of reinfection. UTIs cause significant morbimortality in elderly patients and lead to increased frailty, creating a deleterious vicious circle. Despite some risk factors may not be modifiable, attention should be paid to improve management of urinary incontinence, judicious catheterisation, and antibiotic therapy.

## Supplementary Information

Supplementary Information 1.

Supplementary Information 2.
