# Supplementary material for: Prospective cohort study on hospitalised patients with suspected urinary tract infection and risk factors por multidrug resistance
Source: Sci Rep. 2021 Jun 7;11:11927. doi: 10.1038/s41598-021-90949-2 (PMC8184807; doi:10.1038/s41598-021-90949-2)
Supplement: Supplementary file 1 — Supplementary Information 1. [file 41598_2021_90949_MOESM1_ESM.doc]

Supplementary information:

STROBE statement

Epidemiology, treatment, outcomes, and risk factors for multidrug resistant organisms in hospitalised patients with suspected urine tract infection: a prospective cohort study

**Running head:** Urinary tract infection cohort

Victor Garcia-Bustos^1*^; Ana Isabel Renau Escrig^1^; Cristina Campo López^1^; Rosario Alonso Estellés^1^; Koen Jerusalem^2^; Marta Dafne Cabañero-Navalón^1^; Victoria Morell Massó^3^; Ignacio-Antonio Sigona-Giangreco^5^, José Miguel Sahuquillo-Arce^5^, Iván Castro Hernández^1^; Miguel Salavert Lleti^4^

^1^: Department of Internal Medicine, University and Polytechnic Hospital La Fe, Valencia, Spain

^2^: Department of Internal Medicine, Manises Hospital, Manises, Spain

^3^: Department of Internal Medicine, General Hospital La Mancha Centro, Alcázar de San Juan, Ciudad Real, Spain

^4^: Infectious Disease Unit, University and Polytechnic Hospital La Fe, Valencia, Spain

^5^: Department of Microbiology, University and Polytechnic Hospital La Fe, Valencia, Spain

STROBE Statement—Checklist of items that should be included in reports of ***cohort studies***

|  | Item No | Recommendation |
| --- | --- | --- |
| **Title and abstract** | 1 | (*a*) Indicate the study’s design with a commonly used term in the title or the abstract: Lines 3-5 |
|  |  | (*b*) Provide in the abstract an informative and balanced summary of what was done and what was found: Lines 33-54 |
| Introduction | | |
| Background/rationale | 2 | Explain the scientific background and rationale for the investigation being reported: Lines 57-92 |
| Objectives | 3 | State specific objectives, including any prespecified hypotheses: Lines 93-96 |
| Methods | | |
| Study design | 4 | Present key elements of study design early in the paper: Lines 97-107 |
| Setting | 5 | Describe the setting, locations, and relevant dates, including periods of recruitment, exposure, follow-up, and data collection: Lines 97-107 |
| Participants | 6 | (*a*) Give the eligibility criteria, and the sources and methods of selection of participants. Describe methods of follow-up: Lines 109-141 |
|  |  | (*b*) For matched studies, give matching criteria and number of exposed and unexposed: Not applicable |
| Variables | 7 | Clearly define all outcomes, exposures, predictors, potential confounders, and effect modifiers. Give diagnostic criteria, if applicable: Lines 110-141 |
| Data sources/ measurement | 8* | For each variable of interest, give sources of data and details of methods of assessment (measurement). Describe comparability of assessment methods if there is more than one group: Lines 110-141 |
| Bias | 9 | Describe any efforts to address potential sources of bias: Lines 110-188 |
| Study size | 10 | Explain how the study size was arrived at: Lines 97-100, 110-113 |
| Quantitative variables | 11 | Explain how quantitative variables were handled in the analyses. If applicable, describe which groupings were chosen and why: Lines 172-179 |
| Statistical methods | 12 | (*a*) Describe all statistical methods, including those used to control for confounding: Lines 172-188 |
|  |  | (*b*) Describe any methods used to examine subgroups and interactions: Lines 172-178 |
|  |  | (*c*) Explain how missing data were addressed: Lines 172-188, Tables 1-3 |
|  |  | (*d*) If applicable, explain how loss to follow-up was addressed: not applicable |
|  |  | (*e*) Describe any sensitivity analyses: not applicable |
| Results | | |
| Participants | 13* | (a) Report numbers of individuals at each stage of study—eg numbers potentially eligible, examined for eligibility, confirmed eligible, included in the study, completing follow-up, and analysed: Lines 191-197 |
|  |  | (b) Give reasons for non-participation at each stage: not applicable |
|  |  | (c) Consider use of a flow diagram: not applicable |
| Descriptive data | 14* | (a) Give characteristics of study participants (eg demographic, clinical, social) and information on exposures and potential confounders: Lines 191-212, Table 1 and 2 |
|  |  | (b) Indicate number of participants with missing data for each variable of interest: See Table 1, 2 and 3 |
|  |  | (c) Summarise follow-up time (eg, average and total amount): Lines 266-287 |
| Outcome data | 15* | Report numbers of outcome events or summary measures over time: Lines 266-287 |
| Main results | 16 | (*a*) Give unadjusted estimates and, if applicable, confounder-adjusted estimates and their precision (eg, 95% confidence interval). Make clear which confounders were adjusted for and why they were included: Tables 4, 5 and 6 |
|  |  | (*b*) Report category boundaries when continuous variables were categorized: not applicable |
|  |  | (*c*) If relevant, consider translating estimates of relative risk into absolute risk for a meaningful time period: not applicable |
| Other analyses | 17 | Report other analyses done—eg analyses of subgroups and interactions, and sensitivity analyses: not applicable |
| Discussion | | |
| Key results | 18 | Summarise key results with reference to study objectives: Lines 306-318 |
| Limitations | 19 | Discuss limitations of the study, taking into account sources of potential bias or imprecision. Discuss both direction and magnitude of any potential bias: Lines 346-351, 388-396 |
| Interpretation | 20 | Give a cautious overall interpretation of results considering objectives, limitations, multiplicity of analyses, results from similar studies, and other relevant evidence: Lines 319-384 |
| Generalisability | 21 | Discuss the generalisability (external validity) of the study results: Lines 319-364 |
| Other information | | |
| Funding | 22 | Give the source of funding and the role of the funders for the present study and, if applicable, for the original study on which the present article is based: not applicable |

*Give information separately for exposed and unexposed groups.

**Note:** An Explanation and Elaboration article discusses each checklist item and gives methodological background and published examples of transparent reporting. The STROBE checklist is best used in conjunction with this article (freely available on the Web sites of PLoS Medicine at http://www.plosmedicine.org/, Annals of Internal Medicine at http://www.annals.org/, and Epidemiology at http://www.epidem.com/). Information on the STROBE Initiative is available at http://www.strobe-statement.org.
