# Supplementary material for: Prospective cohort study on hospitalised patients with suspected urinary tract infection and risk factors por multidrug resistance
Source: Sci Rep. 2021 Jun 7;11:11927. doi: 10.1038/s41598-021-90949-2 (PMC8184807; doi:10.1038/s41598-021-90949-2)
Supplement: Supplementary file 2 — Supplementary Information 2. [file 41598_2021_90949_MOESM2_ESM.docx]

**COMORBIDTY AND FUNCTIONAL INDEPENDENT SCALES: QUESTIONNAIRES**

1. **CHARLSON COMORBIDITY INDEX (CCI)**

| **Variable** | **Definition** | **Points** |
| --- | --- | --- |
| **Age** | - | - |
| <50 years | - | 0 |
| 50-59 years | - | 1 |
| 60-69 years | - | 2 |
| 70-79 years | - | 3 |
| ≥80 years | - | 4 |
| **Myocardial infarction (MI)** | History of definite or probable MI (EKG changes and/or enzyme changes) | 1 |
| **Congestive heart failure** | Exertional or paroxysmal nocturnal dyspnoea and has responded to digitalis, diuretics, or afterload reducing agents | 1 |
| **Peripheral vascular disease** | Intermittent claudication or past bypass for chronic arterial insufficiency, history of gangrene or acute arterial insufficiency, or untreated thoracic or abdominal aneurysm (≥6 cm) | 1 |
| **Cerebrovascular accident or transient ischemic attack** | History of a cerebrovascular accident with minor or no residua and transient ischemic attacks | 1 |
| **Dementia** | Chronic cognitive deficit | 1 |
| **Chronic obstructive pulmonary disease** | - | 1 |
| **Connective tissue disease** | - | 1 |
| **Peptic ulcer disease** | Any history of treatment for ulcer disease or history of ulcer bleeding | 1 |
| **Mild liver disease** | Mild = chronic hepatitis (or cirrhosis without portal hypertension). Not to be assigned if moderate-to severe liver disease present | 1 |
| **Uncomplicated diabetes** | Not to be assigned if diabetes with end-organ damage is present | 1 |
| **Hemiplegia** | - | 2 |
| **Moderate to severe chronic kidney disease** | Severe = on dialysis, status post kidney transplant, uraemia, moderate = creatinine >3 mg/dL (0.27 mmol/L) | 2 |
| **Diabetes with end-organ damage** | - | 2 |
| **Localized solid tumour** | - | 2 |
| **Leukaemia** | - | 2 |
| **Lymphoma** | - | 2 |
| **Moderate to severe liver disease** | Severe = cirrhosis and portal hypertension with variceal bleeding history, moderate = cirrhosis and portal hypertension but no variceal bleeding history | 3 |
| **Metastatic solid tumour** | - | 6 |
| **AIDS** | - | 6 |

**Regression equation:**

$\boldsymbol{10-year survival=}\boldsymbol{0.983}^{\boldsymbol{e}^{\boldsymbol{CCI*0.9}}}$

1. **BARTHEL INDEX FOR ACTIVITIES OF DAILY LIVING**

|  | **0 points** | **5 points** | **10 points** | **15 points** |
| --- | --- | --- | --- | --- |
| **Feeding** | Unable | Needs help | Independent | - |
| **Bathing** | Unable | Independent | - | - |
| **Grooming** | Unable | Independent | - | - |
| **Dressing** | Unable | Needs help | Independent | - |
| **Bowel control** | Incontinence (or needs to be given enemas) | Occasional accident | Continent | - |
| **Bladder control** | Incontinence (catheterized and unable to manage alone) | Occasional accident | Continent | - |
| **Toilet use** | Unable | Needs help | Independent | - |
| **Transfers (bed to chair and back)** | Unable | Needs major help (one or two people, physical), can sit | Needs minor help (verbal or physical) | Independent |
| **Mobility on level surfaces** | Immobile or <50 yards | Wheelchair independent, including corners, >50 yards | Walks with help of one person (verbal or physical) >50 yards | Independent (but may use any aid, e.g., stick) >50 yards |
| **Stairs** | Unable | Needs help (verbal, physical, carrying aid) | Independent | -- |

**Barthel index interpretation:**

80-100: independent

60-79: minimally dependent

40-59: partially dependent

20-39: very dependent

<20: completely dependent
